# Prevalence and Correlates of Benzodiazepines' Prescriptions in an Inpatient Setting

**DOI:** 10.1192/bjo.2022.349

**Published:** 2022-06-20

**Authors:** Nada Zahreddine, Rahna Theruvath Chalil, Sohail Abrar

**Affiliations:** Norfolk and Suffolk NHS Foundation Trust, Ipswich, United Kingdom

## Abstract

**Aims:**

The National Institute for Health and Care Excellence guidelines state that benzodiazepines (BZD) should not be taken for longer than four weeks. However, there are no recommendations specifically addressing the use and misuse of BZD in inpatient settings and their prescription at discharge. A recent study (Panes et al., 2020) recommended aiming for BZD’ total withdrawal or, at least, dose reduction at discharge to reduce the risk of misuse in the community which can lead to dependence and serious side effects. Our study aimed to 1. describe BZD’ prescriptions on an acute female ward, before admission, during admission, at the time of discharge and at four and eight weeks post-discharge, 2. identify potential sociodemographic, clinical and therapeutic correlates/predictors of BZD’ prescriptions, 3. develop a strategy to reduce BZD’ prescriptions or, at least, to reduce the dose of BZD prescribed at discharge.

**Methods:**

Data collection was done retrospectively through electronic medical and prescribing records and included admissions to Avocet Ward, between May and October 2021. Variables collected were age, ethnicity, length of stay, Mental Health Act status, diagnosis, comorbid drugs or alcohol misuse, Home Treatment Team involvement at discharge, community teams, prescriptions of regular and Pro Re Nata BZD and “z-drugs” prior to admission, during admission, at discharge, and at 4 weeks and 8 weeks post-discharge, maximum dose of regular BZD during admission and the dose at discharge.

**Results:**

Among the 59 admissions included, 25.4% had BZD before admission, 81.4% during admission (with a mean maximum dose of regular BZD of 38.8 mg (SD = 17.3) of diazepam equivalent), 50.8% at discharge (with a mean dose of 28.5 mg (SD = 18.5) of regular BZD), 35.6% 4 weeks post-discharge and 27.1% 8 weeks post-discharge. The odds of having regular BZD during admission were 7.4 times more likely for those on regular BZD before admission after controlling for other variables (95%CI: 1.1, 50). The maximum dose of regular BZD during admission was positively correlated with the dose of regular BZD at discharge (r(15) = .67, p < .01). Among the regular BZD prescribed during admission (N = 23), 26.1% were fully discontinued by the time of discharge and 43.5% were titrated down, while 30.4% remained at the same maximum dose prescribed during admission.

**Conclusion:**

BZD prescriptions are common at discharge from inpatient settings and can be associated with BZD misuse in the community. We suggest strategies to avoid this issue.

